# WTO, an ontology for wheat traits and phenotypes in scientific publications

**DOI:** 10.5808/GI.2020.18.2.e14

**Published:** 2020-06-16

**Authors:** Claire Nédellec, Liliana Ibanescu, Robert Bossy, Pierre Sourdille

**Affiliations:** 1 Paris-Saclay University, INRAE, MaIAGE, F-78350 Jouy-en-Josas, France; 2 Paris-Saclay University, INRAE, UMR MIA-Paris, AgroParisTech, F-75005, Paris, France; 3 University Clermont-Auvergne, INRAE, UMR 1095 GDEC, F-63000 Clermont-Ferrand, France

**Keywords:** ontology, text mining, wheat trait and phenotype

## Abstract

Phenotyping is a major issue for wheat agriculture to meet the challenges of adaptation of wheat varieties to climate change and chemical input reduction in crop. The need to improve the reuse of observations and experimental data has led to the creation of reference ontologies to standardize descriptions of phenotypes and to facilitate their comparison. The scientific literature is largely under-exploited, although extremely rich in phenotype descriptions associated with cultivars and genetic information. In this paper we propose the Wheat Trait Ontology (WTO) that is suitable for the extraction and management of scientific information from scientific papers, and its combination with data from genomic and experimental databases. We describe the principles of WTO construction and show examples of WTO use for the extraction and management of phenotype descriptions obtained from scientific documents.

**Availability:** WTO ontology (https://doi.org/10.15454/1.4382637738008071E12) is available on AgroPortal: http://agroportal.lirmm.fr/ontologies/WHEATPHENOTYPE under the license Creative Commons Attribution International 4.0 International (CC BY 4.0); The wheat trait bibliographic search engine SAMBlé AlvisIR is available at: http://bibliome.jouy.inra.fr/demo/wheat/alvisir/webapi/search.

## Introduction

Improvement of most animal and plant species of agronomical interest has become an international stake because of the increasing demand for feeding a growing world population. The new environmental constraints such as the reduction of inputs (water, fertilizers, and pesticides) and the reduction of acreages involve the development of new breeding schemes that must be shorter and more powerful. This requires a significant improvement of the agronomical potential of the species through breeding. This is especially true for bread wheat (*Triticum aestivum* L.) which is the most widely grown crop worldwide.

The recent advent of genomic tools contributed to a better understanding of the biological mechanisms underlying the expression of phenotypes of agronomical interest. The availability of genetic information linked to genotyping and phenotyping experimental data obtained from fields and controlled environments has never been greater for understanding biological mechanisms and hypothesizing new models of plant biology [1].

As a consequence, reusing data from different platforms that are obtained through different methods, sensors and protocols, has become a major challenge. The standardization of the information for semantic interoperability of heterogeneous datasets is a key milestone [2]. An ontologie, as defined in [3], is designed to represent the knowledge from one domain by concepts (or classes), relationships among these concepts and instances of these concepts. Therefore ontologies have long been identified as a critical tool for managing information systems in the fields of integrative plant biology, genetics [4]: among others Gene Ontology [5,6] defines gene functions, biological processes and cellular components ; the Plant Ontology (PO) Database [7,8] developed by the Planteome Project is a community resource for plant structure and developmental stages controlled vocabulary and annotations [9]. PO links plant anatomy, morphology and growth and development to plant genomics data.

Dedicated ontologies focus on controlled vocabulary for the description of the phenotypic information. The Plant Trait Ontology (TO) [10] of the Planteome project [11] defines general phenotypic traits in plants. Each trait is a distinguishable feature, characteristic, quality, or phenotypic feature of a developing or mature plant independently of the species. The Crop Ontology (CO) [12,13] is developed by several centers of the Consultative Group on International Agricultural Research (CGIAR) Biodiversity and their partners (Elixir, INRAE, iBET). This ontology focuses on the documentation of phenotype observations as variables that are grouped in nine high-level trait classes. The variables are triplets of observation methods, units of measurement, and traits that encompass the observed entity (e.g., grain, plant). CO distinguishes specific traits for 31 economically important plant species. Their vocabularies have reached different stages of development, ranging from pearl millet (52 variables) to wheat, the richest, with 498 variables.

Beside observation and experimental data, scientific literature is a significant source of genetic and phenotypic information on plants [14,15]. Automatic information retrieval and information extraction have been acknowledged as major challenges in Life Science for assisting manual biocuration, either to assess experimental or inferred data quality or to fill databases with complementary information [16,17]. However, most work focuses on molecular biology, functional and comparative genomics resource development, and phenotypic-related human health, as the Biocreative Track III interactive text mining task in 2012 [18].

In the plant biology domain, information extraction from text has attracted less attention [19], even though the quality and the abstraction of the textual information confer it a significant value for breeding. General properties of plant cultivars as described in the literature are of great interest for many research and innovation studies that are complementary to the detailed and partially unrelated phenotypic observations. Scientific literature summarizes, synthetizes, abstracts and explains experimental results, filtering out spurious observations and highlighting important outcomes. As such it constitutes a valuable source of knowledge for the interpretation of phenotyping experimental results, as well as for the design of plant system biology models able to explain, predict, or simulate genotypic-phenotypic relationships.

**Fig. 1.**
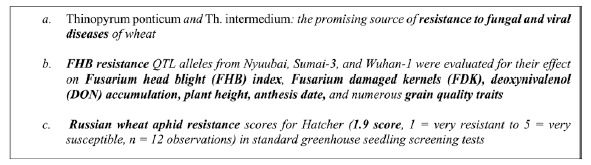
Examples of phenotype mentions from scientific papers. Traits and phenotypes are in bold.

Information extraction from text requires the establishment of dedicated ontologies and of text mining pipelines as largely recognized in the biomedical domain [20]. Ontologies improve text mining performances and conversely the information extracted is more reusable when linked to a reference resource such as an ontology through the normalization process. Normalization consists in assigning a class or a category from a controlled vocabulary to text mentions. It is a key step for the semantic interoperability of textual information and other sources of data and a major text mining challenge [21]. Plant traits and phenotypes expressed in textual sources are characterized by a great variability of the lexicon [15]. The text carries information at various levels of generality with different assessment status, ranging from experimental fine-grained data to general expert knowledge, through intermediate levels of synthesis and abstraction. The examples in Fig. 1 illustrate the variability of trait expressions in scientific documents in descending order of generality. In example (1) the trait “resistance to fungal and viral diseases” is a general trait. Example (2) mentions “FHB resistance” (i.e., Fusarium head blight resistance) which reflects resistance to a specific fungal disease FHB, and its effect on the related observations of six specific traits (e.g., plant height). Example (3) is the most specific: it is about the severity score of the trait “Russian wheat aphid resistance” observed for a given cultivar (i.e., Hatcher) whose value is 1.9.

This varying scope of phenotypic information in scientific papers answers to different needs and usages. It ranges from detailed documentation of experiments and inferred data, to review of shared and well-acknowledged bodies of knowledge supported by large sets of experimental and scientific results.

We have been developing the Wheat Trait Ontology (WTO) since 2010 to answer breeders and scientists’ needs for wheat trait and phenotype information management and retrieval at varying abstraction scales. WTO supports two objectives: (1) building a formal shared representation of wheat trait whose knowledge organization closely reflects the expert knowledge model and (2) making phenotypic information extraction from text easier. To achieve both objectives, the sources for building WTO include expert knowledge and textual documents: expert interviews, terminology analysis from the literature and gene catalogs. The richness of the WTO vocabulary, its similarity with scientific literature lexicon and its deep hierarchies make it a useful resource for both text mining and information management.

The paper is organized as follows. Section 2 describes the WTO. Section 3 presents the motivation and method for building WTO. Section 4 illustrates WTO usage through an application. Section 5 discusses WTO characteristics compared to other semantic resources and presents future work.

## WTO Description

WTO covers a wide range of bread wheat traits (e.g., observable physical plant properties), phenotypes (e.g., trait values) and their related environmental conditions (e.g., disease, extreme temperature) organized in three trees. The current version contains 596 classes. The population of the main classes and their subclasses is given in Table 1.

The maximum depth of WTO is 9 and the average number of children per class is 3. We chose a deep and balanced structure because the breeder's needs to manage data at different levels of aggregation. Classes at intermediate levels support synthetic queries for searching high-level correlations between genetic, phenotypic, and physiological phenomena.

The classes of the ‘Trait’ subtree are linked to the corresponding phenotypes by the “Trait_has_value” relationship. For instance, ‘ear emergence time’ trait class is linked to the ‘late heading’ pheno-type class.

**Fig. 2.**
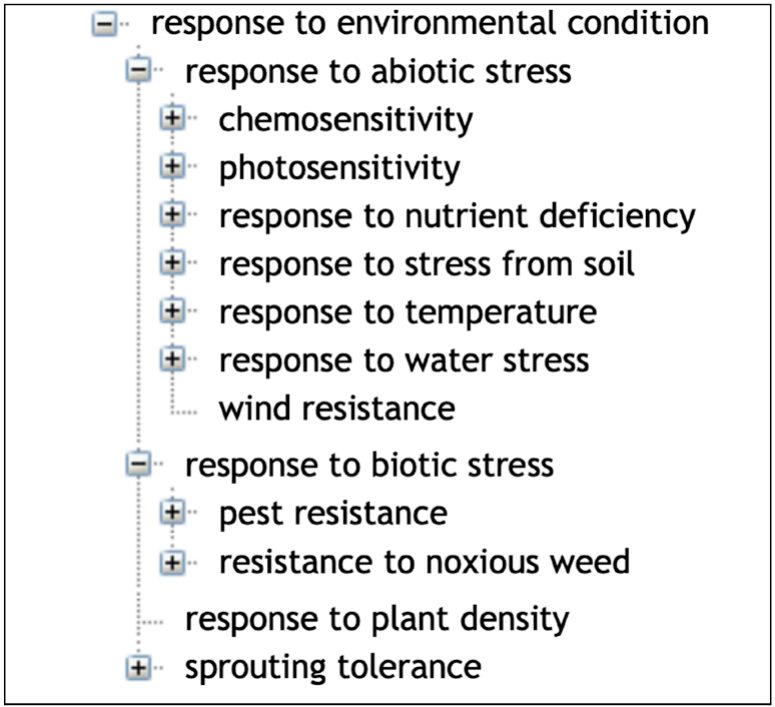
Wheat Trait Ontology (WTO) subclasses for ‘response to environmental condition’.

The ‘Environmental condition’ subtree mainly represents abiotic conditions and biotic conditions that are linked to the corresponding responses of the plant to abiotic stresses and biotic stresses. The main root classes of ‘Response to environmental conditions’ range from response to chemical, radiation, temperature, to a large range of responses to biotic stresses as shown in Fig. 2.

Response to biotic stress is indeed a major concern for wheat breeding. Wheat is affected by several microbial, bacterial, viral and mainly fungal diseases that cause major crop loss [22]. WTO accounts for this situation with two large subtrees ‘Disease’ (58 classes) and ‘Pest’ (103 classes) of ‘Environmental condition’ (Table 1). The relation ‘Causes’ between the ‘Pathogen’ classes and the infectious “Disease” classes represents the causative link between the agent and the disease. WTO distinguishes between disease of bacterial, viral and fungal causal agents. A total of 55 different fungal species causing 44 diseases is described in WTO.

**Fig. 3.**
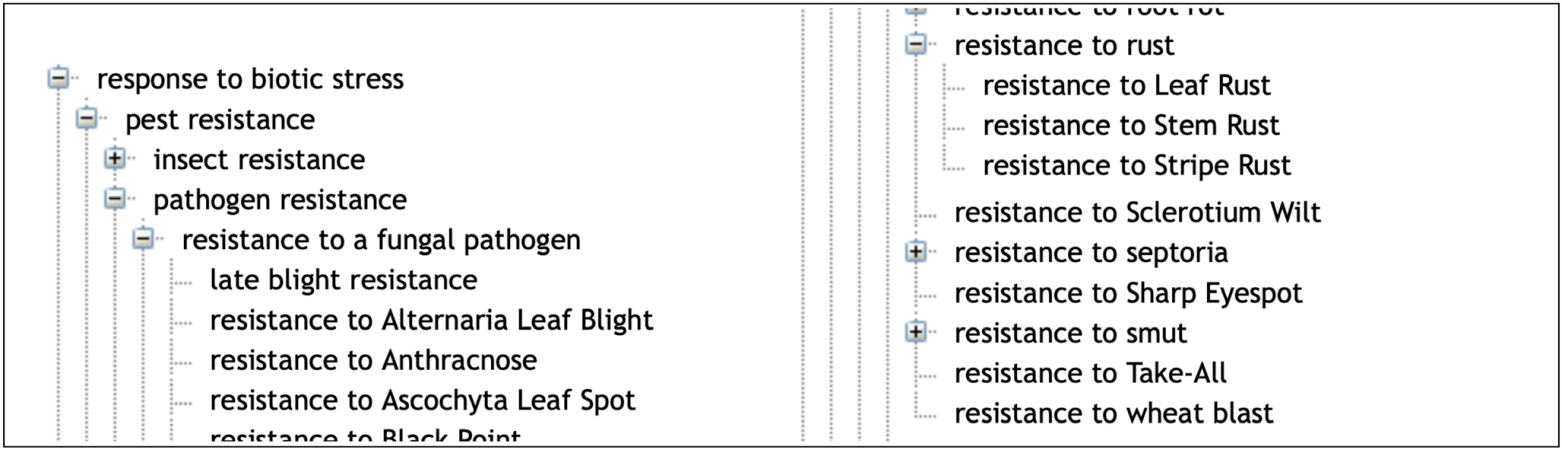
An excerpt of the different ‘resistance to a fungal pathogen’ in Wheat Trait Ontology (WTO).

**Fig. 4.**
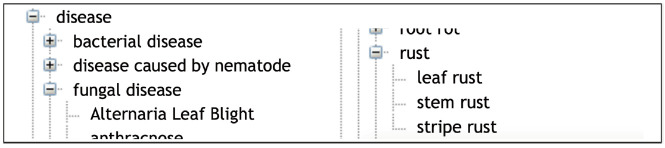
Example of the rust disease family in Wheat Trait Ontology (WTO).

In a similar way, the ‘Response to biotic stress’ subtree finely distinguishes between the causal stress factors as shown in Figs. 3 and 4.

**Fig. 5.**
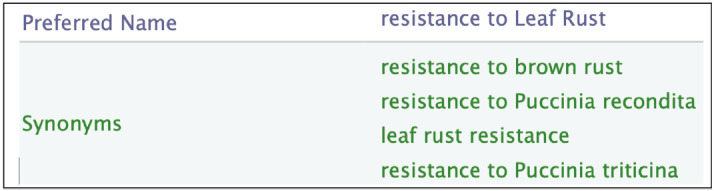
Synonyms of the ‘Resistance to Leaf Rust’ label in Wheat Trait Ontology (WTO). Leaf Rust disease is caused by different fungi, namely ‘Puccinia recondita’ and ‘Puccinia tricina’.

The responses of the plant to biotic stresses are expressed in two ways: either by the disease name or by the causative agent names. A given disease name may have synonyms and a disease may be caused by more than an agent (Fig. 5).

**Fig. 6.**
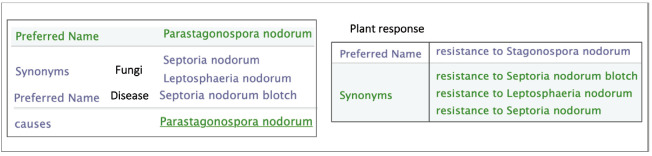
Example of various names of *Parastagonospora nodorum* fungus in Wheat Trait Ontology (WTO).

Moreover, fungi naming in scientific paper do not always strictly follows the nomenclature standard imposed by the mycologist community. For instance, names corresponding to different life stages can be found. For each resistance trait, the causative agents are given with their standard names and the other names as in Fig. 6.

WTO lexical synonymy relations and conceptual relations are complementary with respect to the intended uses to reflect expert knowledge model and make phenotypic information extraction from text easier.

**Fig. 7.**
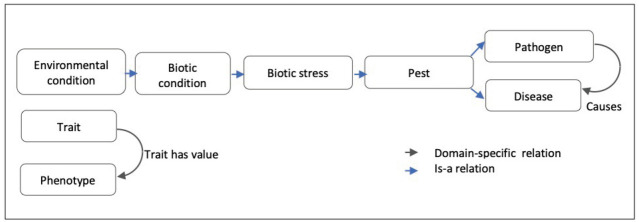
Wheat Trait Ontology (WTO) relations.

As summarized by Fig. 7 WTO structure is mainly hierarchical with two transversal relations: a domain-specific causal one and a variable-value relation.

## Wheat Trait Ontology Building

WTO was built using the NeOn Methodology [23], a scenario-based methodology that supports the collaborative aspects of ontology development and reuse. The WTO development process followed successively Scenario 1, From specification to implementation, and Scenario 2, Reusing non-ontological resources of the NeOn methodology. The first step is to specify the ontology requirements, provided in the next subsection. Then we present insights and rationales for design choices in the following subsection.

### Ontology Requirements Specification

The needs for the development of shorter and more powerful breeding schemes is a strong motivation for sharing phenotypic information linked to genes of interest and traits. Building an open and shared database for marker-based assisted selection (MAS) in bread wheat was the SAMblé project objective (2010–2014) [24]. The SAMBlé database should support both short-term MAS-related goals of breeders, the intended users, and long-term research goals of researchers on underlying biological mechanisms of phenotypes. The information considered for the database was the existence of links between one or more markers and genes of agronomic interest in bread wheat. The information sources were the scientific literature, gene catalogs and in-field and high-throughput phenotyping experiments.

**Fig. 8.**
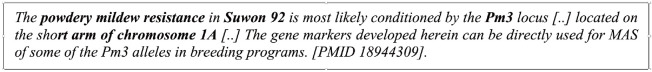
Example of marker in a cultivar related to disease resistance phenotype.

In scientific papers, phenotypic information is frequently linked to varieties, genes or markers and traits as in Fig. 8, which makes it extremely relevant for breeding [15].

This information was first automatically extracted from the literature, then assessed against reference material and elite material (335 varieties) under field conditions for different traits of interest. Finally, the markers that gave the best results and could be used in breeding selection schemes were recorded in the database to be queried by the partner breeders [24]. The traits considered in the SAMBlé project were related to four main large topics, namely, disease resistance, resistance to abiotic stress, plant development, and baking quality.

Representative queries of the breeders were, “which alleles and markers are involved in resistance to rust (e.g., leaf rust, stripe rust, stem rust)” and “what are the varieties tested.” Same question arises for “bread making quality (e.g., flour quality, color, composition, mechanical property, crumb firmness)?”.

A general objective of SAMBlé was to develop a shared database with the information collected by the project that would be easily searchable. The WTO was designed to support this goal. The ontology should support queries on traits and phenotypes at various levels of aggregation combinied with other criteria on markers, genes, and varieties.

To this purpose we created WTO as deep non-strict hierarchies of traits, phenotypes, and environment factors. Non-strict means here that one concept may have several direct parents forming a direct acyclic oriented graph. It covers the large set of topics of the SAMBlé database, ranging from development or resistance to stress to food quality.

### Design and implementation

The design of WTO followed a top-down approach where the core model was first established based on project partner expertise on wheat phenotyping: the SAMblé project gathered breeders from French breeding companies, the French union of breeders (UFS) and Arvalis, it was led by the research unit GDEC-INRAE (Genetics, Diversity and Ecophysiology of Cereals). Text mining and plant information management were provided by Mathematics, Informatics and Genomics Laboratory, French National Research Institute for Agriculture, Food and the Environment (MIG-INRA) and Unité de Recherche Génomique Info, French National Research Institute for Agriculture, Food and the Environment (URGI-INRAE). The main classes of the WTO core model were similar as presented in Table 1 of Section 2. The core model was then extended by reusing information from three external sources: scientific literature, the Catalog of Gene Symbols for Wheat [25] and GrainGene database [26]. The biotic stress response, diseases, and pathogen WTO subtrees (see Section 2) were then significantly restructured by wheat disease experts. We adopted the Obo-Edit tool as ontology editor, to make it easier for biologists and breeders to revise and enrich WTO, compared to more powerful but less user friendly tools.

#### Scientific literature as a source of concepts

An Ontology Acquisition approach was first used to extract and conceptualize WTO concepts and relationships from scientific text expressed in natural language, following the same methodology as described in Nedellec et al. study [27].

We applied the term extractor BioYateA [28] to a scientific corpus to automatically extract relevant domain-specific terms. BioYatea's strength over other term extractors is the ability to extract prepositional phrases that are frequent in wheat trait terms, e.g., response *to* vernalization, florets *without* grain [29]. The scientific corpus was composed of the abstracts and titles of articles. They were obtained from the Web of Science (WoS) bibliographical search engine with the keywords ‘*wheat* or Triticum aestivum and *marker* and *gene*’. It yielded 3,170 references (see Nedellec et al. study [15] for more details).

The candidate terms extracted by BioYateA were then used to derive concepts using the Terminology Design Interface (TyDI) tool. TyDI supports term collaborative assessment and structuring [27]. First, relevant terms were selected among candidate terms by manual screening. Validated terms were grouped in semantic classes of preferred terms, synonyms and typographic and acronym variations. They were structured in hypernym hierarchies consistent with the core model. Concepts and concept hierarchies were then derived from these semantic classes and hypernym trees to populate the core model. The preferred terms were kept as concept labels. This literature term analysis approach sped-up the discovery process of a very large set of trait, phenotype, disease, and pathogen related concepts and subsumption relationships.

#### Other external sources of wheat trait terms

To identify complementary relevant trait terms, we also used the Catalog of Gene Symbols for Wheat (WGC) [30] available online at the Wheat Genetics Resources Database of Japan as a PDF file at the date of WTO building in 2011. The main contribution to WTO from the catalog was related to plant morphology (e.g., plant height) and physiology (e.g., response to photoperiod).

The GrainGenes [26] database was also used for the study of biotic stress response. GrainGenes is a comprehensive resource for molecular and phenotypic information for wheat maintained by U.S. Department of Agriculture and mirrored by MaIAGE. GrainGenes web pages listed general traits and specific traits for wheat, barley, and oat species from which we identified some wheat disease names and their pathogen agents. INRAE experts of wheat diseases then controlled the naming because for some diseases American vernacular naming was not consistent with European naming.

### WTO evolution

**Fig. 9.**
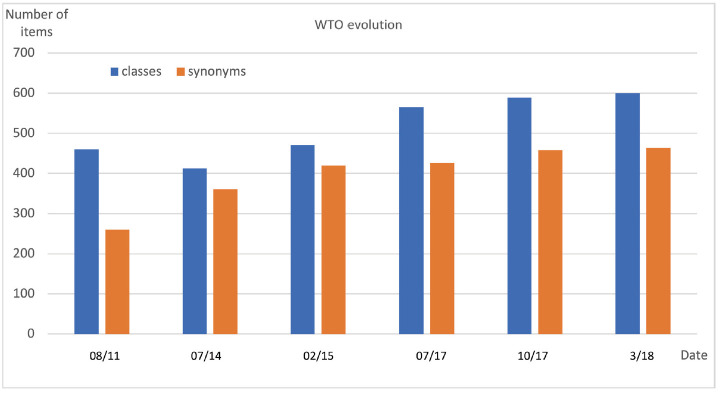
Statistics of the Wheat Trait Ontology (WTO) between 2010 and 2020.

Fig. 9 displays the evolution of the WTO (formerly named Wheat Phenotype Ontology) between 2010 and 2020. The first public version of WTO was released in August 2011. It contained 460 classes and 260 synonyms of labels.

The 2011 version of WTO was revised in 2014. Confusions between pathogen names and synonyms were corrected which resulted in an increased number of synonyms and decreased number of classes. In 2015, the classes ‘Fiber quality’, ‘Food property’, ‘Milling quality’, ‘Grain composition’ and ‘Grain quality’ were grouped in a new ‘Quality’ class in order to reduce the number of root classes and to increase WTO readability. Conversely the ‘Development’ class that mixed phenological phenotypes, morphology (e.g., color, length) and growth (related to yield) was split into three distinct classes: ‘Development’, ‘Morphology’ and ‘Growth’.

In 2017, with the purpose of using WTO for managing other phenotypic databases than the SAMBlé one, we evaluated WTO scope with respect to two external resources. The WIPO (the Wheat INRA Phenotype Ontology formerly named the INRA Wheat Ontology) [31] developed by URGI-INRAE and the “list of wheat descriptors for Characterization and Evaluation” of the NARO GeneBank project. A few more morphology terms such as ‘presence of awn’, ‘glume pubescence’, ‘glume color’ were then added to WTO.

### WTO for marker-assisted selection

To be used as the conceptual formalization of the SAMblé database schema, WTO was integrated into the MAS (Marker Assisted Selection) knowledge model detailed in Nedellec et al.'s study [15]. The MAS model was designed to manage the entities and relations of the SAMBlé database. It contains 8 entity types and 14 n-ary relationships for the representation of the genotypic and phenotypic information and relationships collected from the literature and experiments of the SAMBlé project. The main MAS model entities are ‘Marker’, ‘Type’, ‘Allele’ and ‘Gene’, 'Trait' and ‘Phenotype’ and ‘Variety’. ‘Type’ represents the type of method used to identify the marker, e.g., amplified fragment length polymorphism, microsatellite. The main relationships are ‘Marker tags Gene in Variety’ between markers, genes and varieties, ‘Trait has Phenotype in Variety’ between traits, phenotypes and varieties and ‘Gene expresses Phenotype in Variety’ between genes, phenotypes, and varieties.

The connection of the MAS model to WTO is achieved through the straight forward alignment of two pairs of MAS and WTO classes: (1) the MAS 'Trait' class is aligned with WTO ‘Plant property’ class, the root of the trait subtree (2) the MAS 'Phenotype' class is aligned with WTO ‘Phenotype’ class, the root of WTO phenotype subtree. The other MAS classes (e.g., Gene, Marker, Variety) are also connected to nomenclatures and catalogs (e.g., Genes nomenclatures, Markers lists, and Variety catalogs) for data standardization. The integrated MAS and WTO model was successfully used for the management of SAMBlé database information and for information extraction from text.

## Wheat Trait Ontology Usage

WTO has been validated through the use by breeders and researchers involved in the SAMBlé project of two end-user applications, the SAMBlé database interface [24] and the Wheat literature semantic search engine AlvisIR. AlvisIR supports queries on genes, varieties, markers, phenotypes and traits extracted from PubMed references. Phenotype and trait expressions in text are normalized by WTO concepts.

**Fig. 10.**
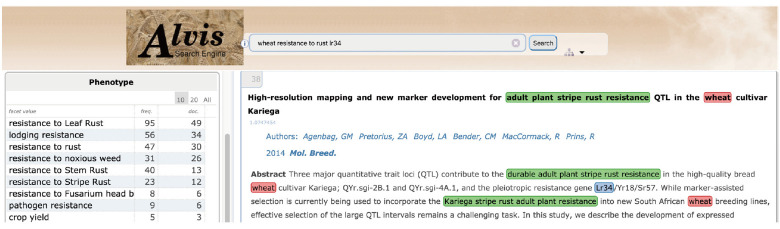
Screenshot of AlvisIR semantic search engine query web page.

Fig. 10 gives an example of a semantic search for phenotypic information. The example query asks for documents where the gene ‘Lr34’ is mentioned in relation to the trait ‘resistance to rust' in 'wheat' by combining the three keywords, ‘lr34’, ‘resistance to rust’, and ‘wheat’. The first hit displays a document extract where ‘adult plant stripe rust resistance’ (underlined in green) is tagged by the query ‘resistance to rust’ keyword.

**Fig. 11.**
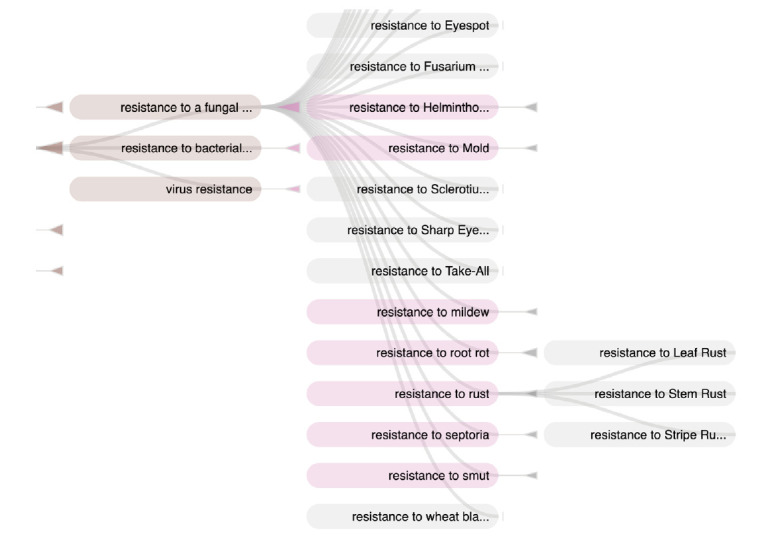
Screenshot of ontology navigation in AlvisIR semantic search engine.

'Resistance to rust' in the user query has been interpreted by three complementary mechanisms. A text mining workflow run in batch mode has first automatically extracted all terms from the documents, among which the term ‘adult plant stripe rust resistance’, and automatically mapped it to the relevant WTO class ‘Resistance to stripe rust’. The query interpreter executed on the fly has segmented the user query and mapped the query term ‘resistance to rust’ to the corresponding WTO class. The subsumption relation between the query class and the document class has then been verified. The document term is therefore validated as an instance of the query term and the document is displayed as a hit. Fig. 11 shows the corresponding subpart of WTO with the two mapped classes. A navigation tool (Fig. 11) supports the expression of the query by the user by the combination of selected classes. The users from the SAMBlé project are satisfied with the balanced and deep tree structure of WTO that makes ontology browsing and class selection much easier than a flat and large list of classes would.

High-level queries as exemplified here are powerful for combining criteria on phenotypes with other genetic or environmental information as requested by the SAMBlé project.

The online version of AlvisIR indexes PubMed abstracts. PubMed has been preferred over WoS for its Open Access license to references. Current work includes the extension of the corpus to full papers of main scientific journals. Eighteen thousand papers have been identified among which half are available through Open Access and 1,361 journals targeted. The text mining workflow named WheatLiterature used to fill in the database from the scientific literature is based on the AlvisNLP technology (AlvisNLP on Github). It is distributed as a component of the European text mining OpenMinTeD platform [32].

## Discussion

Beyond semantic search, ontology-based fine-grained information extraction is a key component of the integration of textual information with experimental and genetic data. However, the reference knowledge models often differ with the sources and the nature of the information. Their alignment and user query rewriting are a major challenge for data integration [33].

Significant work has been done on Wheat Data Interoperability Guidelines [34] that focuses on Minimum Information About a Plant Phenotyping Experiment (MIAPPE) [2,35]. For experimental data, observation variables including traits but also observation protocol, unit of measure and development stage are critical for properly documenting the observations and determining if observations are comparable or not. This leads to building trait ontologies as WIPO [31] or Crop Ontology [13] where the trait leaves are database variable traits (e.g., ‘Susceptibility to leaf rust' in WIPO,’ Leaf rust severity’ in Crop Ontology). Phenotypes, the values of the traits, (e.g., ‘*Susceptible* to leaf rust’) are not conceptualized as classes but represented by the database numerical data as values of the trait variable. For instance, in WIPO the trait Disease intensity score takes values on a 1 to 9 increasing scale (1, no disease; 9, very severe). Such ontologies are suitable for accurately documenting observations and for the computation of correlations by statistical tools.

Conversely, WTO aimed at managing both traits and phenotype values represented by expressions, as they occur in the scientific literature. For instance, in the *the leaf rust susceptible cultivar ‘GA 100’* phrase, the phenotype value is *‘leaf rust susceptible’* and the variety is *‘GA 100’*. In this way, WTO representation then supports SAMblé data discovery by direct queries on traits and phenotype values (e.g., ‘Leaf rust susceptibility’) at various levels of generality (e.g., ‘Rust susceptibility’, ‘Fungal disease susceptibility’) and their relation to other information (e.g., cultivars).

Similar queries on observation databases that follow MIAPPE recommendations would require the translation of numerical values by using value domains or thresholds, i.e., discretization and hierarchization of the phenotypes. Moreover, the lack of depth of ontologies such as WIPO or Crop Ontology with a comb-like structure does not allow high-level queries. An example in WIPO, is the trait ‘Susceptibility to leaf rust’, which is a direct subclass of the high level ‘biotic stress trait’ without intermediate levels. Similarly, in the Crop Ontology the trait ‘Fusarium head blight AUDPC’ is a direct subclass of ‘biotic stress trait’.

Another representative example is ‘Nitrogen harvest index’. In WIPO and Crop Ontology, it has only one direct ancestor, which is ‘Quality trait’, with 51 other sibling traits in Crop Ontology. In WTO, ‘Nitrogen harvest index’ has five successive ancestors: ‘Nitrogen use efficiency’, ‘Macronutrient use efficiency’, ‘Nutrient use efficiency’, ‘Growth’ by increasing order of generality.

The integration of the two sources of data, observations, and synthetic information from text in a same data management system should preserve the best of the two approaches. It would require the alignment of the ontology classes and the rewriting of the phenotype variable values to map them to qualitative descriptors.

In the SAMBlé project and for development of the OpenMinTeD Wheat use case, we experienced this situation with the two ontologies: WIPO, which indexes experimental phenotype data, and WTO, which indexes PubMed phenotypic information. Their classes are not mappable in a straight-forward one-to-one way. It is noteworthy that the types of alignments and rewriting identified during these projects are not specific to wheat or even to plants, but are general to any phenotype observation data. Further investigation of this question is a future challenge for the integration of phenotype data from different sources allowing a better exploitation of textual data.

## Conclusion

We proposed WTO, a reusable ontology of bread wheat traits and phenotypes and related environmental factors. The design of the model relies both on domain expert knowledge and the analysis of evidence published in the scientific literature. The WTO model is deeply structured, well reflecting the domain knowledge. It facilitates navigation and reuse for data and knowledge discovery. The model was designed to support the extraction and the management of marker-assisted selection information. WTO is also a contribution to the description of the link between genetic and phenotypic information. Concept synonyms were directly extracted from the literature, which turns WTO a suitable resource for Information Extraction and Information Retrieval. WTO has been assessed for its consistency through its use. WTO is complementary to other ontologies dedicated to the documentation of phenotypic observations. We believe that future work on their alignment and mapping will favor data semantic interoperability from the literature and experimental sources.

## Figures and Tables

**Table 1.** Main classes of WTO with the number of some subclasses

	No.
Environmental condition	221
Abiotic condition (e.g., chemical, nutrient, water, wind)	51
Biotic condition	171
Biotic stress	170
Disease	58
Bacterial disease	6
Fungal disease	44
Viral disease	6
Diseased caused by nematode	2
Pest	103
Insects	21
Plant property	374
Phenotype	45
Trait	326
Development (plant habit, precocity, vernalization)	19
Growth (crop yield, nutrient use efficiency, density)	41
Morphology (of awn, glume, grain, spike)	23
Quality	58
Food property	30
Grain composition	12
Grain quality	13
Milling quality	4
Reproduction	5,173
Response to environmental conditions	64
Response to abiotic stress	104
Response to biotic stress	

WTO, Wheat Trait Ontology.
